# TrioVis: a visualization approach for filtering genomic variants of parent–child trios

**DOI:** 10.1093/bioinformatics/btt267

**Published:** 2013-05-08

**Authors:** Ryo Sakai, Alejandro Sifrim, Andrew Vande Moere, Jan Aerts

**Affiliations:** ^1^Department of Electrical Engineering-ESAT, KU Leuven, SCD-SISTA, Leuven 3001, Belgium, ^2^Future Health Department, iMinds, Leuven 3001, Belgium and ^3^Department ASRO, KU Leuven, Research[x]Design, Leuven 3001, Belgium

## Abstract

**Summary:** TrioVis is a visual analytics tool developed for filtering on coverage and variant frequency for genomic variants from exome sequencing of parent–child trios. In TrioVis, the variant data are organized by grouping each variant based on the laws of Mendelian inheritance. Taking three Variant Call Format files as input, TrioVis allows the user to test different coverage thresholds (i.e. different levels of stringency), to find the optimal threshold values tailored to their hypotheses and to gain insights into the global effects of filtering through interaction.

**Availability:** Executables, source code and sample data are available at https://bitbucket.org/biovizleuven/triovis. Screencast is available at http://vimeo.com/user6757771/triovis.

**Contact:** ryo.sakai@esat.kuleuven.be

## 1 INTRODUCTION

Recent advances in massively parallel sequencing technologies, especially sequencing of the entire protein-coding portion of the genome (exome), have introduced new strategies for identifying Mendelian disease genes (Gilissen *et al.*, 2012). Analysis of parent–child trios is one of the strategies for identifying single pathogenic mutations among the thousands to millions of genomic variants. By sequencing, the patient as well as his or her parents, variants can be filtered based on consistency or inconsistency according to the laws of Mendelian inheritance.

Although filtering based on inheritance pattern seems to be straight-forward, distinguishing true variation from artefacts and false negatives while retaining sensitivity is a challenging task because of the sequencing error rate and the interdependency of sequencing quality for multiple samples. A previous study (Bamshad *et al.*, 2011) reported that >70% of Mendelian inconsistencies were found to be false negatives because of the failure to call the germ line variant in either parent sample in search for *de novo* mutations. Similarly, we found that the majority (77%) of variants were consistent with the Mendelian laws when we analysed those variants that are in common between the exome sequencing and an SNP genotyping array for a trio-case (data not shown). One of the metrics commonly used to filter variants is the depth of coverage. Researchers we interviewed adjust the coverage threshold based on the overall coverage and their intuition without any visual aids. The optimal coverage thresholds also depend on other factors, such as the suspected type of mutation, whether somatic or inherited, and the stringency of analysis. Although finding the optimal coverage threshold can be automated to some extent, it still requires fine adjustments of the filtering setting for variant discovery.

We present a visual analytics tool, TrioVis, designed to help the analytical reasoning process of setting coverage thresholds to filter variants from parent–child trio sequencing experiments. It visualizes variants in a structured table and provides interactive visual interfaces to let the researcher dynamically and interactively test different threshold settings and change levels of stringency.

## 2 FEATURES

TrioVis is a stand-alone, desktop application developed in Processing (Reas *et al.*, 2007), an open source programming language and integrated development environment (IDE), based on Java, and is available for Linux, Mac OS X and Windows. It loads three separate Variant Call Format (VCF) files, and sample VCF files were generated using the GATK Unified Genotyper (DePristo *et al.*, 2011). It requires the AD (Depth Per Allele By Sample) field, which includes the unfiltered count of reference (REF) or alternative (ALT) reads. Based on these read counts, variant frequencies for each variant are calculated. A sample dataset, generated from the BAM files of Utah residents with Northern and Western European ancestry (CEU) trios from the 1000 Genome Project (The 1000 Genomes Project Consortium, 2010), is available for download.

**Fig. 1.**
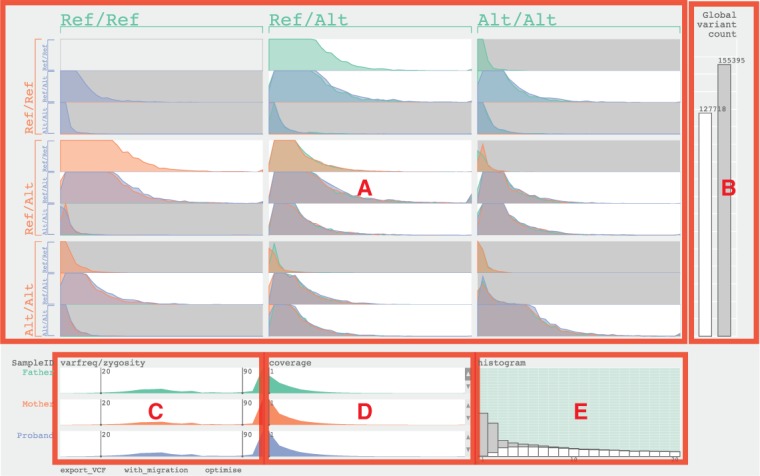
The user interface of TrioVis and five sections labelled (A–E) for parent–child trio data from exome sequencing by Illumina HiSEQ 2000 are used. (**A**) The main table. (**B**) The global variant count bar graphs. (**C**) The variant frequency sliders. (**D**) The coverage sliders. (**E**) The histogram view showing the father sample

The user interface consists of five sections: the main table (Fig. 1A), the global variant count bar graphs (Fig. 1B), the variant frequency sliders (Fig. 1C), the coverage sliders (Fig. 1D) and the histogram view (Fig. 1E). Each section focuses on a specific aspect of trio data and offers specific interactive features to calibrate the thresholds. Father, mother and child are colour-coded in green, orange and blue, respectively.

The *main table* (Fig. 1A) is divided into small multiples based on the pattern of inheritance. Each block consists of three histograms, conveying the distribution of variants based on the read depth per sample. The background colour of each block is determined by whether it is consistent (white) or inconsistent (grey) with the laws of Mendelian inheritance. The *global variant count bar graphs* (Fig. 1B) represents the total counts of variants based on whether it is consistent. By changing coverage settings, the researcher aims to minimize the number of inconsistent calls while keeping the number of consistent calls high.

The *variant frequency sliders* (Fig. 1C) visualize the distribution of variants based on variant frequency values. These sliders can be used by the researchers to adjust the ranges for variant frequency for genotyping variants for that sample. By default, any variants with variant frequency >90 are considered alternative homozygous, and any variants with variant frequency between 20 and 89 are considered alternative heterozygous. Any variants <20 are filtered out. The *coverage sliders* (Fig. 1D) set the coverage thresholds for each sample individually. These sliders also represent the distribution of variants based on coverage values. Finally, the *histogram view* (Fig. 1E) represents the distribution of consistent and inconsistent variants in stacked bar graphs with coverage values between 1 and 20 for the selected sample. Hovering the mouse over the stacked bar graph highlights cells in the main table, showing where these variants are represented. This view aids the researcher to calibrate the coverage threshold for the selected sample.

The variant data can be investigated under two assumptions: with the ‘migration’ assumption, any variant below the coverage threshold is considered homozygous reference; when this assumption is inactive, variants below the coverage threshold are considered invalid and discarded from the combined set of variants. Filtered results can be exported using the ‘export VCF’ button and saved as VCF files. The researcher can also select specific blocks to export variants of a specific inheritance pattern (i.e. *de novo* mutations and recessively inherited variants) for further analysis. The ‘optimize’ function finds the best-weighted average of the precision and recall (f-score) based on the number of filtered consistent and inconsistent variants, providing the user a good initial setting for further investigation and adjustment.

## 3 CONCLUSION

TrioVis provides an interactive interface and optimization function to calibrate coverage thresholds based on Mendelian inheritance laws for parent–child trio cases. By visualizing variants in a novel table layout based on the inheritance laws, it allows the researcher to gain insights into the global effect of filtering in the context of trio analysis. The researcher can export the filtered result as VCF files for subsequent analysis to annotate variants to genes, using annotation tools, such as Annotate-It (Sifrim *et al.*, 2012) and Annovar (Wang *et al.*, 2010). Future work includes improving the optimization algorithm, and integration of this tool into functional annotation tools, such as Annotate-It and Galaxy (Goecks *et al.*, 2010).
